# Lipofection with Synthetic mRNA as a Simple Method for T-Cell Immunomonitoring

**DOI:** 10.3390/v13071232

**Published:** 2021-06-25

**Authors:** Natalia Teresa Jarzebska, Julia Frei, Severin Lauchli, Lars E. French, Emmanuella Guenova, Cécile Gouttefangeas, Thomas M. Kündig, Mark Mellett, Steve Pascolo

**Affiliations:** 1 Department of Dermatology, University Hospital Zürich (USZ), University of Zürich (UZH), Raemistrasse 100, 8091 Zürich, Switzerland; NataliaTeresa.Jarzebska@usz.ch (N.T.J.); Julia.Frei@usz.ch (J.F.); Severin.Laeuchli@usz.ch (S.L.); emmanuella.guenova@unil.ch (E.G.); Thomas.Kuendig@usz.ch (T.M.K.); Mark.Mellett@usz.ch (M.M.); 2 Faculty of Science, University of Zürich, 8006 Zürich, Switzerland; 3 Faculty of Medicine, University of Zürich, 8006 Zürich, Switzerland; 4 Department of Dermatology and Allergy, University Hospital, LMU Munich, 80539 Munich, Germany; Lars.French@med.uni-muenchen.de; 5 Dr. Philip Frost, Department of Dermatology and Cutaneous Surgery, University of Miami Miller School of Medicine, Miami, FL 33146, USA; 6 Department of Dermatology and Venereology, Faculty of Biology and Medicine, Lausanne University Hospital (CHUV), University of Lausanne, 1000 Lausanne, Switzerland; 7 Department of Immunology, University of Tübingen, 72074 Tübingen, Germany; Cecile.Gouttefangeas@uni-tuebingen.de

**Keywords:** transfection, ivt mRNA, immunomonitoring, lipofection, TLR7/8, T-cells

## Abstract

The quantification of T-cell immune responses is crucial for the monitoring of natural and treatment-induced immunity, as well as for the validation of new immunotherapeutic approaches. The present study presents a simple method based on lipofection of synthetic mRNA in mononuclear cells as a method to determine in vitro T-cell responses. We compared several commercially available transfection reagents for their potential to transfect mRNA into human peripheral blood mononuclear cells and murine splenocytes. We also investigated the impact of RNA modifications in improving this method. Our results demonstrate that antigen-specific T-cell immunomonitoring can be easily and quickly performed by simple lipofection of antigen-coding mRNA in complex immune cell populations. Thus, our work discloses a convenient solution for the in vitro monitoring of natural or therapy-induced T-cell immune responses.

## 1. Introduction

The intricate complexity of the human immune system makes it challenging to analyze its responses to infection, disease, injury, or medical intervention. Immunomonitoring of T-cell responses provides information on the nature and state of immune reactions and is required to assess the efficacy of a medical treatment or to predict its effects [1]. Phenotyping of immune cell populations aids in the elucidation of the cellular mechanisms underlying newly developed immunotherapeutic approaches. It can also identify the presence of cellular and molecular signatures that categorize patients into distinct risk groups and/or help to predict clinical responses to therapy [2].

For the purpose of T-cell immunomonitoring, autologous target cells expressing epitopes of interest are usually required [3]. Defined epitopes can be introduced into immune cells through various methods, including overlapping peptides [4], viral vectors [5], or via the transfection of plasmid DNA or synthetic in vitro transcribed mRNA (ivt mRNA) coding the epitope or protein of interest [6,7]. 

Among these methods, mRNA is the most promising, as mRNA is easy to produce, and can encode full-length antigens or epitopes either in their wild-type form or in chimeric proteins for specific delivery to antigen presentation compartments [8,9,10].

The most popular strategy for immune response monitoring includes in vitro transfection by electroporation of antigen-presenting cells (APCs), such as dendritic cells (DCs), or of peripheral blood mononuclear cells (PBMCs) [3,10,11]. 

Such methodology based on electroporation is cumbersome and time consuming and is therefore not adequate for high-throughput T-cell immunomonitoring assays. 

To attain a faster, easier, and more robust method for T-cell immunomonitoring, we explored whether freshly-isolated PBMCs could be transfected with mRNA using commercially available transfection reagents, including lipoplexes and polyplexes, to induce mRNA-coded epitope-specific immune responses. We aimed to define simple conditions to induce an immune response using mRNA in nanoparticles. This approach is an easy and straightforward method that can be implemented in high-throughput experiments.

## 2. Materials and Methods

### 2.1. Messenger RNA Preparation

Firefly Luciferase-coding mRNAs were produced using in vitro transcription at the “ivt mRNA production and formulation platform” in Zürich (http://www.cancer.uzh.ch/en/Research/mRNAPlatform.html, accessed on 26 January 2021). The 5′ end consisted of a CleanCap^TM^ (Trilink, San Diego, CA, USA) followed by an eIF4G aptamer as the 5′ untranslated region [12] (that, as we could previously show, enhances translation of both unmodified and pseudouridine-modified ivt mRNA) and by a codon-optimized firefly luciferase open reading frame. The 3′ end consisted of a tandem repeat of the mouse beta globin 3′ UTR and a poly-A tail [13]. The transcription of mRNA was performed in the presence of the four canonical bases (A, C, G, and U) to obtain immuno-stimulatory RNA and in the presence of pseudouridine instead of uridine to obtain immuno-silent mRNA. RNA was diluted in RNase-free water, and the concentration (after quantification using a Nanodrop) was adjusted to 1 mg/mL. The quality and integrity of ivt mRNAs were checked using agarose gel electrophoresis. The mRNAs were stored at −20 °C.

### 2.2. Cell Culture and Luciferase Experiments

Human embryonic kidney (HEK) 293 cells, murine colon carcinoma CT26 cells, and murine melanoma B16-F10 cells were maintained in RPMI medium (ThermoFisher Scientific, Waltham, MA, USA) containing 10% fetal calf serum (FCS), 200 mM l glutamine (Gibco, Waltham, MA, USA), and 0.2% antimicrobial reagent Normocin (InvivoGen San Diego, CA, USA), named complete medium in the following. The following mRNA lipofection agents were used: Lipofectamine Messenger Max (Invitrogen, Waltham, MA, USA), RiboJuice mRNA transfection kit and RiboJuice siRNA transfection kit (Merck Millipore Sigma Aldrich St. Louis, MO, USA), mRNA-Fect (RJH Biosciences Edmonton, AB, Canada), Screenfect (ScreenFect Transfections, Eggenstein-Leopoldshafen, Germany), and JetMessenger (Polyplus Transfection, Illkirch-Graffenstaden, France). For luciferase experiments, transfection of the above-mentioned cell lines was performed with 100,000 cells per well in 200 microliters of complete medium. mRNA carriers were prepared according to manufacturer protocols and added to plated cells to obtain a final mRNA concentration of 1 µg/mL, and incubated with cells for the indicated time. Luciferase activity was recorded one day after transfection by adding 25 microlitres of Bright-Glo luciferase assay solution (Promega, Madison, WI, USA) and measuring the activity using the GloMax Discover and Explorer Detection System equipment (Promega, Madison, WI, USA). 

### 2.3. Measuring the Stimulation of the Innate Immune Response

Blood samples were obtained from healthy donors. Spleens were obtained from BALB/c or C57BL/6 mice housed with ethical approval from the Cantonal Veterinary Office of Zürich, Switzerland (license ZH215/17). Spleens were dilacerated and splenocytes were resuspended in 10 mL of complete medium. For both human and mouse cells, mononuclear cells were isolated using the Ficoll-Paque™ Plus (GE Healthcare Life Sciences, Marlborough, MA, USA) method. A total number of one million cells per well were plated on 96-well plates and incubated overnight with appropriate RNA carrier containing 200 ng of RNA per well (200 μL cultures). Protamine RNA particles were prepared as described previously [14,15]. The next day, supernatants were taken, and IFNα concentrations were measured via ELISA by following the manufacturer’s protocol (Human IFNα pan ELISA development kit, MABTECH, ELISA MAX Standard Set Mouse IFNα, Biolegend, San Diego, CA, USA). The absorbance was measured at 450 nm with an ELISA reader (GloMax Discover and Explorer Detection System equipment, Promega, Madison, WI, USA) and cytokine concentrations were calculated according to a standard curve.

### 2.4. Measuring the Stimulation of Adaptive Immunity in Murine Cells

C57BL/6-Tg(TcraTcrb)1100Mjb (22) mice, also referred to as OT1 mice, were a generous gift from Pål Johansen (University Hospital Zurich). Spleen mononuclear cells from OT1 mice were isolated using the Ficoll-Paque™ Plus (GE Healthcare Life Sciences, Marlborough, MA) method. A total number of 200,000 cells per well (200 μL culture) were plated on 96-well plates and incubated overnight with appropriate mRNA carrier containing 200 ng of immunostimulating mRNA per well. The next day, supernatants were taken, and interleukin-2 (IL-2) concentration was measured via ELISA, as per the manufacturer’s protocol (ELISA MAX Standard Set Mouse IL-2, Biolegend, San Diego, CA, USA). The absorbance was measured at 450 nm with an ELISA plate reader (GloMax Discover and Explorer Detection System equipment, Promega, Madison, WI, USA).

### 2.5. Measuring the Stimulation of Adaptive Immunity in Human Cells

Blood samples were obtained from healthy, HLA-A2-positive donors. Mononuclear cells were isolated using the Ficoll-Paque™ Plus (GE Healthcare Life Sciences, Marlborough, MA, USA) method. A total number of 1 × 10^6^ cells per well were plated on 24-well plates and incubated with the appropriate mRNA carrier containing 200 ng of each indicated mRNA per well (1 mL cultures). Starting day 4, every other day of the culture, 10 U/mL IL-2 (R&D Systems, Minneapolis, MN, USA) was added to the cells. Cells were cultured for 12 days. At day 12, cells were stained with phycoerythrin (PE)-HLA-A*0201 tetramers containing the conserved immunodominant HLA-A*0201 epitope from influenza matrix M1 [16], at a final concentration of 5 μg/mL in PBS (The Tetramer Shop, Kongens Lyngby, Denmark) during 15 min at room temperature. Then, the cells were stained with a fluorescein isothiocyanate (FITC)-conjugated anti-CD3 antibody and a peridinin–chlorophyll–protein complex (PerCP)-conjugated anti-CD4 antibody (Becton Dickinson, Heidelberg, Germany) in a PBS buffer supplemented with 0.5% bovine serum albumin and 2 mM EDTA for 30 min at 4 °C. The cells were washed twice with PBS before being fixed with 1% formaldehyde and analyzed by flow cytometry (LSRForstessa, BD Biosciences, New Jersey, NJ, USA). 

## 3. Results

### 3.1. Screening of an Optimal Transfection Agent for Primary Immune Cells 

We tested six different commercially available transfection agents (Table 1), four lipoplexes (MessengerMax, RiboJuice siRNA reagent, RiboJuice miRNA reagent, and Screenfect) and two polyplexes (mRNA-Fect and JetMessenger), for their capacity to transfect murine splenocytes and human PBMCs (in addition to both human and murine cancer cell lines). The transfection proficiency was determined by measuring luciferase activity 24 h post luciferase-coding ivt-mRNA lipofection, upon the addition of luciferin substrat.

**Figure 1:**
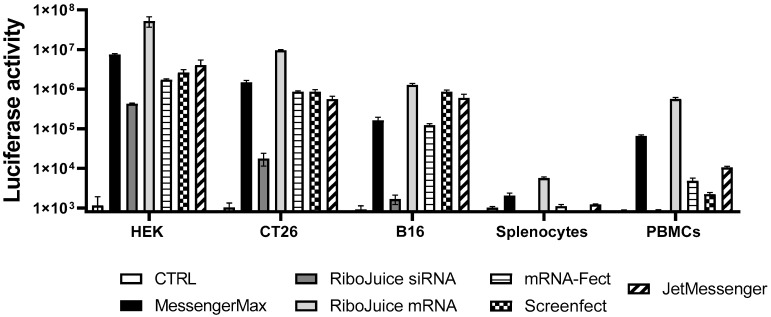
Transfection efficiency of various commercially available transfection reagents. Established human and murine cell lines were transfected with 20 ng mRNA coding for firefly luciferase, whereas primary human PBMCs and murine splenocytes were transfected with 100 ng mRNA. These doses were found as optimal with regard to transfection efficiency and toxicity (Figure S1). Non-transfected cells of each cell type served as the negative control group (CTRL). Luciferase activity was measured 24 h post transfection in a white 96-well plate. Data represent triplicate mean values; error bars: SD.

All the tested formulations could efficiently transfect tumor cells (Figure 1 and summary in Table 1). RiboJuice siRNA was the least efficacious in all cells, whereas RiboJuice mRNA demonstrated the highest transfection efficiency. In splenocytes and PBMCs, RiboJuice mRNA again displayed the best transfection efficacies. The lipoplex JetMessenger and the liposomal MessengerMax also provided robust expression of luciferase in hard-to-transfect primary cell populations. For the purpose of further experiments, we chose MessengerMax, RiboJuice mRNA, and JetMessenger, as they were the most efficient in transfecting mononuclear cells. Our results complement the previous finding that MessengerMax was superior to ScreenFect in transfecting human primary macrophages with ivt mRNA [17].

### 3.2. Effect of the Carrier on the Capacity of mRNA to Stimulate an Innate Immune Response

We next evaluated the ability of mRNA encapsulated in these chosen carriers to induce an innate immune response, by monitoring levels of interferon alpha (IFNα). Foreign RNA can stimulate endosomal Toll-like receptors (TLRs) [18,19]. When triggered, TLRs induce specific intracellular pathways that result in the expression of cytokines, including anti-viral type I interferons [14]. Unmodified single-stranded RNA (ssRNA) is recognized by human TLR7, expressed by plasmacytoid DCs (the main producers of IFNα) and human TLR8, and expressed by monocytes (which are capable of producing large amounts of TNFα) [20].

**Figure 2:**
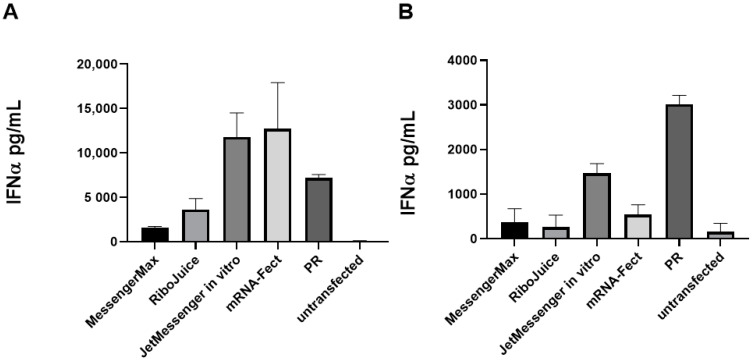
Immune stimulation of mRNA carriers in human PBMCs and murine splenocytes. Cells were seeded at 100,000 cells per well in a 96-well plate and transfected with 200 ng of mRNA per well. Twenty-four hours post transfection supernatants were subjected to ELISA analysis. Selected transfection reagents were tested for their ability to stimulate the production of IFNα in human (**A**) and murine (**B**) immune cells. PR: protamine–RNA nanoparticles. Data represent triplicate mean values; error bars: SD.

All the nanoparticles containing non-modified mRNA are immunostimulatory in vitro and induce IFNα release in murine and human immune cells (Figure 2A,B). In human cells, MessengerMax prompted the lowest production of IFNα, while mRNA-Fect and JetMessenger induced the highest. In murine splenocytes, RiboJuice and MessengerMax induced the lowest stimulation, whereas JetMessenger again induced the highest signal. This was interesting as both RiboJuice and MessengerMax induced the highest luciferase activity in human PBMCs and murine splenocytes (Figure 1), but this did not correlate with the highest innate immune response in these cells. This could be due to the different mechanisms of entry that the formulations illicit, i.e., the polyplex, JetMessenger, mediates cell entry via endocytosis, which could trigger the endosomal TLRs, leading to a greater IFNα response. Indeed, we also used protamine/RNA complexes as a positive control, where the cationic protamine forms nanoparticles with negatively charged RNA. These nanoparticles enter the cells via endosomes and induce elevated levels of IFNα (Figure 2A,B). IFNα stimulation is an important factor in the context of immunomonitoring in vitro, as a strong IFNα response can limit T-cell proliferation [21]; therefore, it may be preferred to prevent the induction of a strong IFNα response in cell cultures. 

### 3.3. Optimising mRNA-Based T-Cell Immunomonitoring In Vitro in Murine Cells

To deter production of IFNα, next we sought to evaluate the effects of immuno-silent ivt mRNA, where uridine was replaced with pseudouridine (as previously described [22] ). Substitution of uridine with pseudouridine in the ivt mRNA diminished the effects of the formulations to induce expression of IFNα (Figure S2).

**Figure 3:**
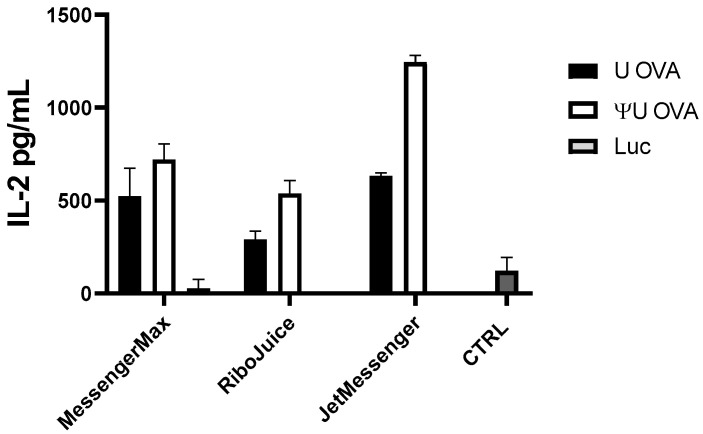
OT1 murine splenocytes were cultivated for 24 h with ovalbumin-coding (OVA) or luciferase-coding (Luc) mRNA. Ovalbumin-coding mRNA induced an adaptive immune response in OT1 cells, which is indicated by elevated IL-2 levels. Non-transfected OT1 cells served as the negative control group (CTRL). Data represent triplicate mean values; error bars: SD.

To test whether particle-based mRNA transfection in immune cells induces detectable adaptive immune responses, we monitored the secretion of interleukin-2 (IL-2) in splenocytes cultured from OT1 mice. OT1 T cells express the transgenic T-cell receptor that recognizes ovalbumin (OVA) peptide. T cells are stimulated when they recognize the OVA peptide SIINFEKL (residues 257–264) presented on the MHC class I molecule, H-2 Kb [23]. As RiboJuice mRNA, MessengerMax, and JetMessenger gave the highest luciferase expression in PBMCs, they were used to transfect splenocytes from OT1 with ivt mRNA-coding ovalbumin. We observed elevated IL-2 levels in cells transfected with all formulations containing ovalbumin-coding mRNA, while particles containing luciferase-coding mRNA did not induce any response in OT1 cells (Figure 3).

Since the induction of type I interferons can limit the proliferation of T cells and synthesis of new proteins, including cytokines, we compared the impact of substituting immuno-stimulating uridine (U) with immuno-silent pseudouridine (ΨU) on the capacity of ivt mRNAs to activate OT1 splenocytes. Indeed, immuno-silent mRNA induced stronger activation of OT1 T cells than immuno-stimulating ivt mRNA (Figure 3). The greatest improvement was observed with JetMessenger, which is consistent with the high induction of IFNα triggered by this polyplex formulation when it contains unmodified mRNA (Figure 2). Immuno-silent ivt mRNA formulated with MessengerMax had little impact on the production of IL-2 compared to immuno-stimulatory ivt mRNA; this is probably due to the low immuno-stimulatory effects of the MessengerMax formulation, which promotes only slight IFNα expression (Figure 2).

Therefore, we were able to establish optimal ivt mRNA formulations for immunomonitoring that displayed limited innate and augmented adaptive immune responses.

### 3.4. mRNA-Based In Vitro Immunomonitoring of Human T-Cells in Response to an Influenza Viral Protein 

We also evaluated our lipofection-based immunomonitoring approach in an ex vivo human system. To that end, PBMCs from an HLA-A*0201 healthy donor were isolated and incubated with formulated mRNAs that encoded the influenza matrix protein M1 (Flu M1) or luciferase as a control. Formulations tested were RiboJuice mRNA and MessengerMax, which induced the greatest expression of luciferase in PBMCs and the lowest induction of IFNα (Figure 1 and Figure 2). IFNα levels were measured in supernatants from all treated groups 24 h post transfection. IFNα could be strongly induced only in groups cultured with immuno-stimulating ivt mRNA and not in groups cultured with immuno-silent mRNA (Figure S3). 

**Figure 4:**
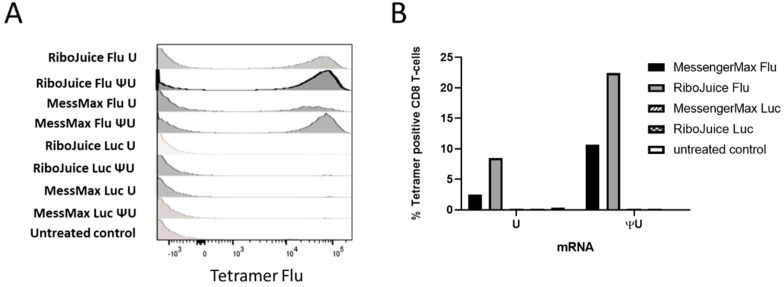
Monitoring of Flu M1-specific immune responses in human PBMCs following transfection with ivt mRNA coding the Flu matrix M1 protein. (**A**) At day 12 of culture, the cells were stained with antibodies and a fluorescent MHC class I tetramer specific for Flu M1 HLA-A*0201-restricted T cells, and analyzed by FACS. (**B**) Comparison of the percentage of Flu M1 MC class I tetramer positive T cells after transfection with immuno-stimulating (“U”) or immuno-silent (“ΨU”) mRNA coding for the Flu M1 HLA-A*0201 epitope.

Next, we tested the expansion of Flu M1-specific T cells in PBMCs transfected with immuno-stimulatory or immuno-silent ivt mRNA with MessengerMax and RiboJuice formulations. RiboJuice mRNA was slightly more efficacious than MessengerMax at promoting an adaptive immune response, as determined by tetramer-specific staining for Flu M1 HLA-A*0201-restricted T cells (Figure 4). Furthermore, the immuno-silent ivt mRNA induces an augmented higher percentage of CD8+ T cells compared with immuno-stimulatory ivt mRNA with both RiboJuice and MessengerMax (Figure 4). Meanwhile, the method was tested using frozen PBMCs from healthy HLA-A*0201 donors (Figure S4). Again, culturing cells in the presence of mRNA coding the Flu matrix M1 protein formulated in MessengerMax allowed the amplification of T cells specific for the immunodominant Flu M1 HLA-A*0201 epitope.

Collectively, these data show that mRNA-transfected PBMCs efficiently expressed and presented the mRNA-encoded Flu M1 epitope to T cells, and that Flu M1-specific T cells proliferated when the PBMCs were transfected with Flu M1 mRNA, but not when transfected with Luc-coding mRNA. These results were further confirmed in two other HLA-A2-positive donors (Figure S4). Complementing findings from the murine system, stimulation of the adaptive immune response was stronger in vitro with immuno-silent RNA, compared to immuno-stimulating RNA: 22.4 versus 8.52% for RiboJuice and 10.7 versus 2.49% for MessengerMax, respectively (Figure 4B). This also corroborates the previous finding that type I interferon prevents proliferation of T cells in vitro [21,24,25]. 

## 4. Discussion

Synthetic mRNA has been successfully used as a vector for induction of antigen-specific immune responses in vitro and in vivo. Immunomonitoring using mRNA transfection in PBMCs is a fast and easy method compared to mRNA transfection in dendritic cells. Currently, mRNA-based immunomonitoring requires electroporation, which although efficacious hinders the adoption of the method, particularly as it requires special equipment and high amounts of mRNA (usually 10 micrograms per electroporation). We demonstrate here that newly available efficacious reagents, i.e., highly functional stabilized mRNA and efficacious transfection reagents, allow us to transfect human primary blood cells and murine splenocytes (Figure 1). We aim to develop mRNA transfection via nanoparticles in PBMCs into a quick and reliable technique for monitoring the antigen-specific T-cell responses present in periphery. With such an approach, we aimed to optimize and achieve a simple and robust method, easily accessible to every laboratory without the need for specialized equipment or excessive time consumption and additionally, with a lower amount of mRNA required (1 microgram per mL).

Among all tested transfection reagents, we found that transfection efficiency depends on cell origin and cell type, and there is no single reagent that would be optimal for all cell types (Table 1 and Figure S5). Although RiboJuice mRNA appears to be globally the most efficacious reagent, it is less stable and has to be used within 5 min of formulation, making it slightly less easy to use than others, including MessengerMax, which is an efficacious, stable, and robust transfection reagent, as it could efficiently deliver mRNA to all tested cell types. During the course of experimentation, we optimized conditions that led to detectable expression levels of the protein encoded by exogenous mRNA in primary mouse and human immune cells. In addition, such mRNA-transfected cells presented MHC class I epitopes to T cells, as documented by the specific activation of SIINFEKL specific mouse T-cells or specific expansion of influenza-specific human T cells upon transfection with antigen-encoding mRNA.

We observed quantitative differences in cytokine release depending on the choice of type of mRNA used in transfection. Unmodified mRNA induced a strong innate immune response in primary cells in all tested conditions; in human PBMCs and murine splenocytes. Meanwhile, mRNA with pseudouridine in its sequence in place of uridine, so-called “immuno-silent” mRNA, did not induce innate immune responses, but better prompted an adaptive immune response, i.e., elevated IL-2 levels in mouse OT1 cells transfected with ovalbumin-coding mRNA (Figure 3) and increased proliferation of human epitope-specific T cells (Figure 4A and Figure S4). It is well established that RNA activates cells of the innate immune system by stimulating Toll-like receptors, specifically TLR3, TLR7, and TLR8 [26,27]. However, when naturally occurring modified nucleosides, for example, pseudouridine, are incorporated into the transcript, the TLRs can no longer be triggered [22]. Studies revealed that incorporating pseudouridine into mRNA not only suppresses RNA-mediated immune activation in vitro and in vivo, but also enhances the translational capacity of the RNA [26]. One cause of this translational difference is that pseudouridine-containing mRNA activates RNA-dependent protein kinase (PKR) less efficiently than uridine-containing mRNA [28]. Another likely contributing factor to the enhanced translation observed with pseudouridine modification is an increase in biological stability of the mRNAs [26]. Indeed, higher resistance to hydrolysis by phosphodiesterases from snake venom and spleen was reported when uridine was replaced with pseudouridine in dinucleotide substrates [29]. Other studies have also demonstrated that pseudouridine stabilizes RNA secondary structures by promoting base stacking, which could slow degradation [30].

Immunomonitoring has become increasingly relevant in the many medical fields. In immuno-oncology it is used for the identification of potential prognostic or predictive immune biomarkers and a better understanding of their underlying mechanisms of action, leading to improved personalized treatments [31]. In addition, the SARS-CoV-2 pandemic that emerged in late 2019 revealed the necessity of a rapid and simple immunomonitoring method. Development of new therapies as well as new pathogen-specific vaccines and treatments require proper assessment of innate and adaptive immune responses, as human immune systems are highly variable [32]. A number of researchers are studying the immune response to SARS-CoV-2 to reveal a systems-level perspective on the immune system changes during the acute and recovery phases of severe COVID-19 disease [33]. We envision that our simplified method for immunomonitoring presented here will be widely implemented to study the T-cell immune responses triggered in patients suffering from infections, cancers, and autoimmunity or inflammatory diseases.

## Figures and Tables

**Table 1:** Summary of the properties of transfection agents used in this paper.

Table	Compound	Transfection Efficiency				
		HEK	CT26	B16F10	Murine Splenocytes	hPBMCs
Messenger Max	Liposome	++	++	+	++	++
RiboJuice mRNA	cationic polymer/lipid mixture	+++	+++	+++	+++	+++
RiboJuice siRNA	cationic polymer/lipid mixture	+	+/−	−	−	−
mRNA-Fect	amphiphilic polymer	+	++	+	+	+
Screenfect	Liposome	++	++	++	−	+/−
JetMessenger	cationic polymer	++	++	++	+	+

## Data Availability

Data is contained within the article.

## Supplementary Materials

The following are available online at https://www.mdpi.com/article/10.3390/v13071232/s1, Figure S1: mRNA dose-dependent (A) transfection efficacy and (B) toxicity in human PBMCs and HEK cells; Figure S2: Differences in innate immune response in murine splenocytes transfected with mRNA containing uridine (U) or pseudouridine (ΨU) in various carriers; Figure S3: IFNα levels in supernatants from hPBMCs 24 h post transfection with indicated mRNA containing uridine (U) or pseudo-uridine (ΨU) in various carriers and the negative control group (CTRL); Figure S4: Monitoring of Flu M1-specific immune responses in human PBMCs following transfection with ivt mRNA coding the Flu matrix M1 protein (Flu) or Luciferase (Luc); Figure S5: Flow cytometry analysis of hPBMCs transfected with different reagents containing ZsGreen-coding mRNA.
