# Supplementary material for: Lipofection with Synthetic mRNA as a Simple Method for T-Cell Immunomonitoring
Source: Viruses. 2021 Jun 25;13(7):1232. doi: 10.3390/v13071232 (PMC8310085; doi:10.3390/v13071232)
Supplement: Supplementary file 1 [file viruses-13-01232-s001.zip › viruses-1231991-supplementary.pdf]

Supplementary Material – viruses-1231991

Lipofection with Synthetic mRNA as a Simple Method for T-Cell Immunomonitoring

Natalia Teresa Jarzebska [1,2], Julia Frei [1,3], Severin Lauchli [1,3], Lars E. French [4,5], Emmanuella Guenova [1,3,6], Cécile Gouttefangeas [7], Thomas M. Kündig [1,3], Mark Mellett [1,3] and Steve Pascolo [1,3,*]

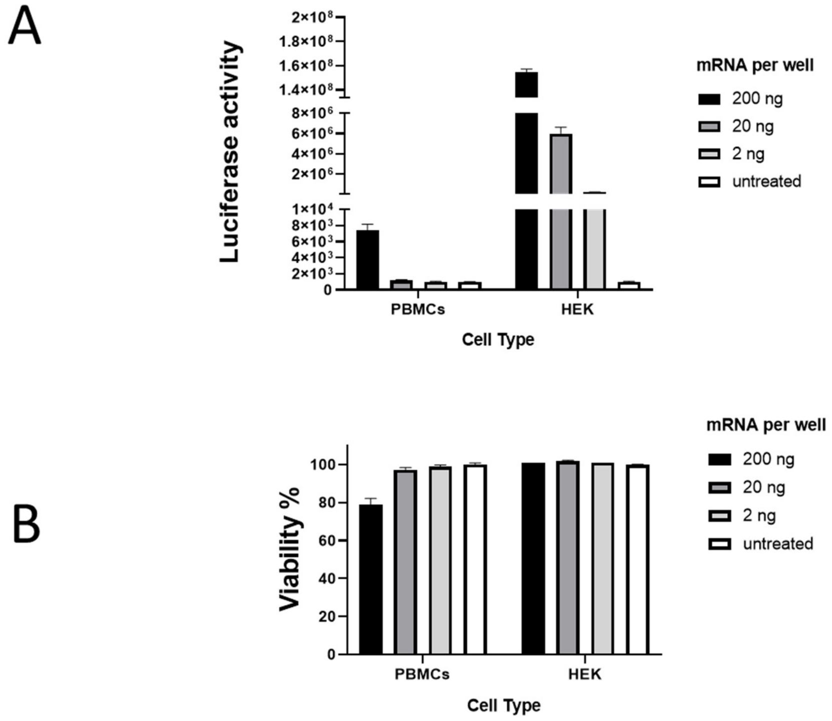

Figure S1. mRNA dose-dependent (**A**) transfection efficacy and (**B**) toxicity in human PBMCs and HEK cells. Cells were transfected with indicated amounts of Luciferase-coding mRNA in
MessengerMax and incubated for 24 h. After that time, supernatants were taken for LDH assay and Bright-Glo luciferase assay solution was added to the wells for luciferase activity measurement. Data represent triplicate mean values; error bars: SD. Viability was measured by LDH Cytotoxicity assay (Promega) using the formula 100* (1-(Experimental-Spontaneous)/(Maximum-Spontaneous)).

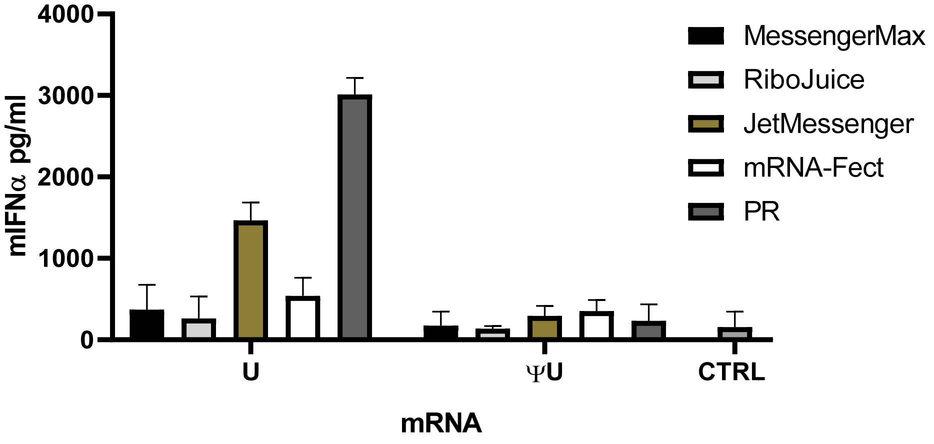

Figure S2. Differences in innate immune response in murine splenocytes transfected with mRNA containing uridine (U) or pseudo-uridine (ΨU) in various carriers and the negative control group (CTRL). Data represent triplicate mean values; error bars: SD. PR: protamine–RNA nanoparticles.

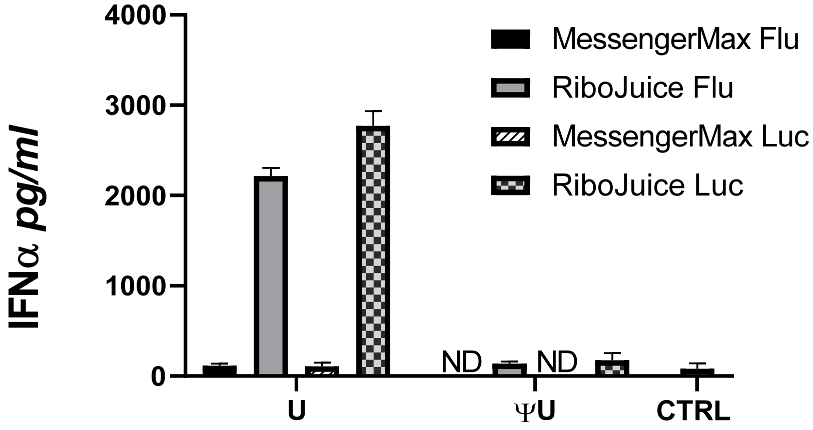

Figure S3. IFNα levels in supernatants from hPBMCs 24 h post transfection with indicated mRNA containing uridine (U) or pseudo-uridine (ΨU) in various carriers and the negative control group (CTRL). Data represent triplicate mean values; error bars: SD; ND: Not Detected.

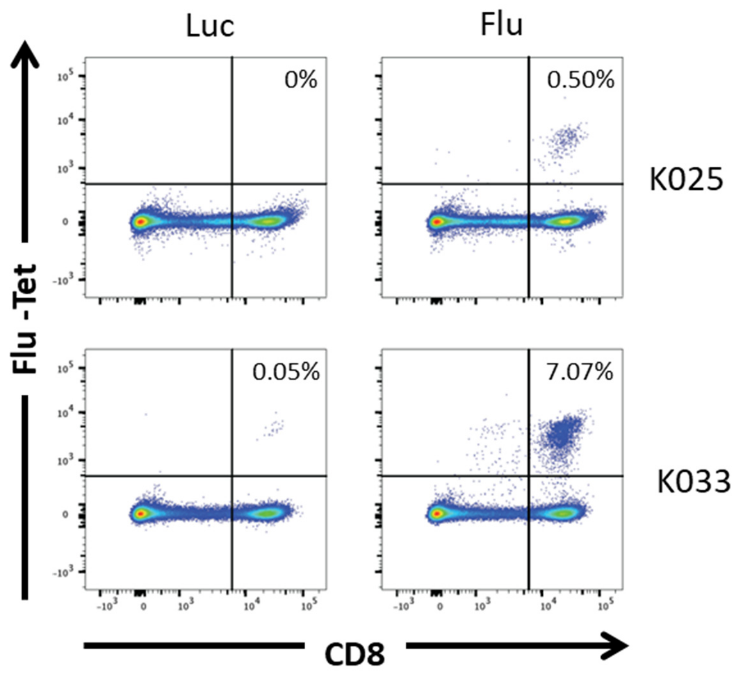

Figure S4. Monitoring of Flu M1-specific immune responses in human PBMCs following transfection with non-modified ivt mRNA coding the Flu matrix M1 protein (Flu) or Luciferase (Luc). Frozen PBMCs obtained from two HLA-A2-positive healthy donors, here called K025 and K033, were cultured, stained, and acquired as described in the Materials and Methods section except that CD8 PE-Cy7 was used to label CD8 cells. Transfection of Flu matrix M 1-coding mRNA gave expansion of Flu M1 tetramer-stained CD8-positive cells.

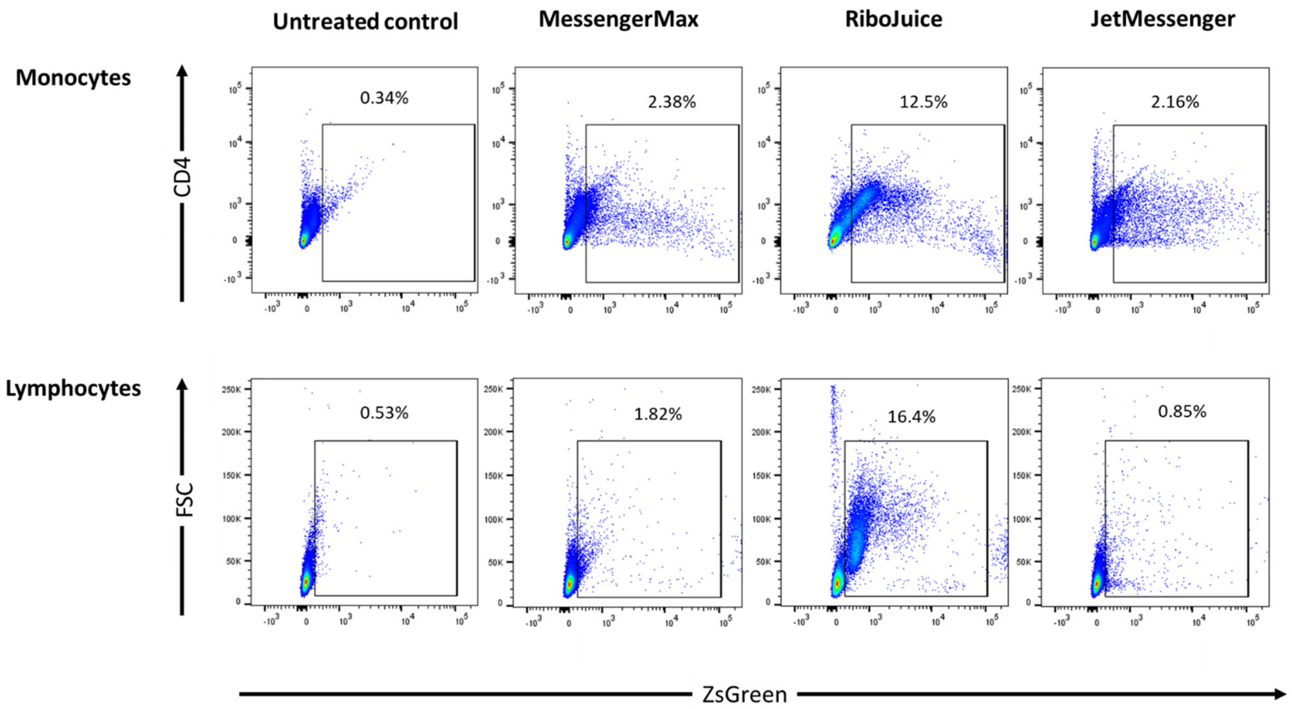

Figure S5. Flow cytometry analysis of hPBMCs transfected using different reagents containing ZsGreen-coding mRNA. The graph shows gated monocytes (CD19 and CD3 double-negative cells) or gated lymphocytes (CD3-positive plus CD19-positive cells). Fresh hPBMCs were obtained from healthy donors and transfected with 1 µg /mL of ZsGreen-coding mRNA. After 24 h of culture, cells were harvested and stained with a PE-Cyanine7-conjugated anti-CD3 antibody, a peridinin–

chlorophyl–protein complex (PerCP)-conjugated anti-CD4 antibody, and allophycocyanin-conjugated anti-CD19 antibody (Becton Dickinson, Heidelberg, Germany) in a PBS buffer supplemented with 0.5% bovine serum albumin and 2 mM EDTA for 30 min at 4°C. The analysis was performed as described in the Materials and Methods section. The results show that all reagents could transfect monocytes and lymphocytes, with Ribojuice outperforming MessengerMax and JetMessenger as was seen using the luciferase readout.
